# DIEP breast reconstruction following multiple abdominal liposuction procedures

**Published:** 2014-12-30

**Authors:** Mohammed Farid, Simon Nicholson, Ashutosh Kotwal, Augustine Akali

**Affiliations:** Department of Plastic Surgery, Hull and East Yorkshire NHS Trust, Hull, United Kingdom

**Keywords:** liposuction, DIEP, free flap, breast reconstruction, angiography

## Abstract

**Objective:** Previous abdominal wall surgery is viewed as a contraindication to abdominal free tissue transfer. We present two patients who underwent multiple abdominal liposuction procedures, followed by successful free deep inferior epigastric artery perforator flap. We review the literature pertaining to reliability of abdominal free flaps in those with previous abdominal surgery. **Methods:** Review of case notes and radiological investigations of two patients, and a PubMed search using the terms “DIEP”, “deep inferior epigastric”, “TRAM”, “transverse rectus abdominis”, “perforator” and “laparotomy”, “abdominal wall”, “liposuction”, “liposculpture”, “fat graft”, “pfannenstiel”, with subsequent appraisal of relevant papers by the first and second authors. **Results:** Patient 1 had 3 episodes of liposuction from the abdomen for fat grafting to a reconstructed breast. Subsequent revision reconstruction of the same breast with DIEP flap was preceded by CT angiography, which demonstrated normal perforator anatomy. The reconstruction healed well with no ischaemic complications. Patient 2 had 5 liposuction procedures from the abdomen to graft fat to a wide local excision defect. Recurrence of cancer led to mastectomy and immediate reconstruction with free DIEP flap. Preoperative MR angiography demonstrated a large perforator right of the umbilicus, with which the intraoperative findings were consistent. The patient had an uneventful recovery and good healing with no fat necrosis or wound dehiscence. **Conclusions:** We demonstrate that DIEP flaps can safely be raised without perfusion-related complications following multiple liposuction procedures to the abdomen. The safe interval between procedures is difficult to quantify, but we demonstrate successful free flap after 16 months.

Poster presentation at the joint conference of British Association of Plastic and Reconstructive Surgeons (BAPRAS) and Irish Association of Plastic Surgeons (IAPS) - November 2013, Dublin, Ireland.

## INTRODUCTION

There are concerns that abdominal surgery can damage the perforator vessels used in deep inferior epigastric artery perforator (DIEP) flaps, leading to compromise of flap viability or reliability. Therefore, any previous surgery involving the abdominal wall is often regarded as a relative contraindication to perforator-based abdominal free tissue transfer.^1^ Determining the reliability of perforators following liposuction from the abdominal wall remains a challenge.

Previous studies investigated the effects of single episode liposuction on the reliability of perforator vessels, focusing largely on transverse rectus abdominis myocutaneous (TRAM) flaps in live patients, and in animal and cadaveric models.^2^ We present two cases of DIEP breast reconstruction following multiple liposuction procedures to the abdominal wall.

## METHODS

Review of case notes and radiological investigations of two patients, and a PubMed search using the terms “DIEP”, “deep inferior epigastric”, “TRAM”, “transverse rectus abdominis”, “perforator” and “laparotomy”, “abdominal wall”, “liposuction”, “liposculpture”, “fat graft”, “pfannenstiel”, with subsequent appraisal of relevant papers by the first and second authors.

## RESULTS

### Case 1

A 57-year-old woman underwent right mastectomy for invasive ductal carcinoma, and immediate reconstruction with latissimus dorsi flap and expandable implant in 2004, followed by adjuvant chemo- and radiotherapy. The patient had no significant medical history, took Anastrozole, and was a non-smoker.

The patient developed capsular contracture and underwent exchange of implant and simultaneous fat transfer (Coleman's technique) in December 2009. 240 mls of fat was harvested from the infra-umbilical abdomen. A second fat transfer in April 2010 harvested 300ml from the same site. Further loss of fat graft volume warranted a third fat transfer in December 2010, with 300 mls harvested from abdomen.

**Figure 1:**
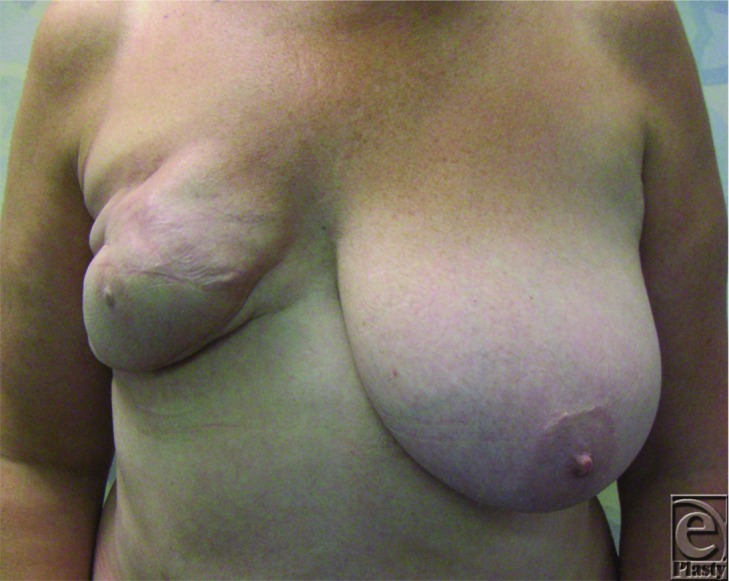
Case 1 - Appearance of previously reconstructed right breast before revision reconstruction with DIEP flap.

Thin skin overlying the implant and a recurrent capsular contracture 16 months later (Fig. 1) led the patient to request removal of implant and DIEP flap reconstruction.

**Figure 2:**
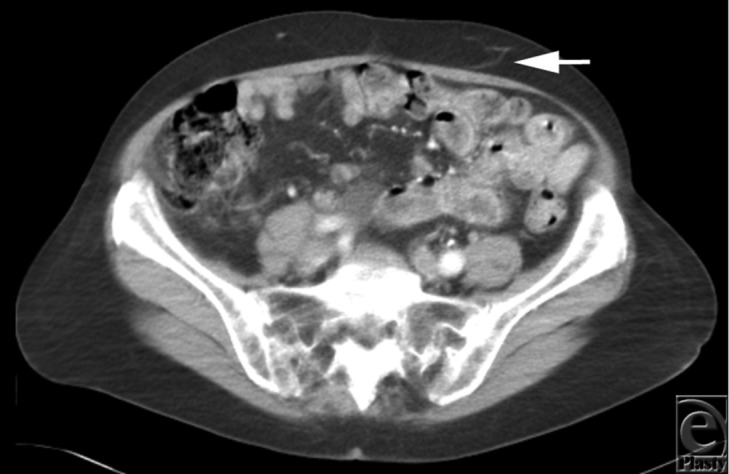
Case 1 - Left sided perforator vessel supplying overlying skin and fat (arrow) on CT angiogram.

**Figure 3:**
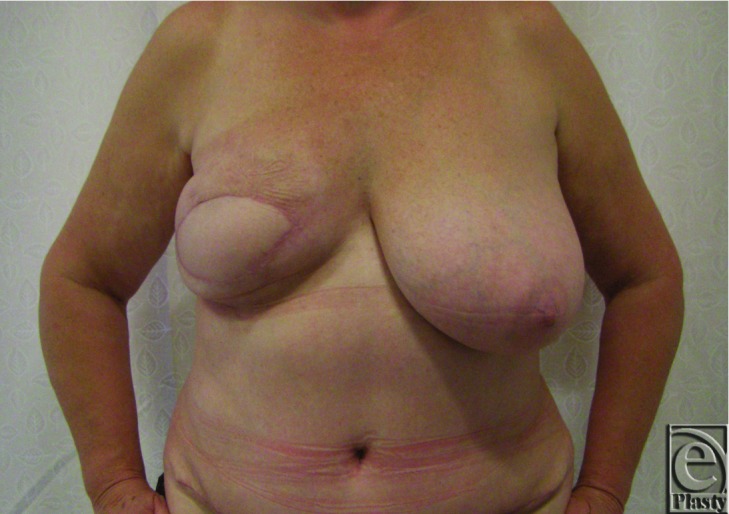
Case 1 - Appearance 5 months following DIEP flap breast reconstruction

Pre-operative CT angiography demonstrated normal-looking abdominal and perforator anatomy (Fig. 2). Intraoperatively, very minimal scarring from the liposuction was noted. A 610g DIEP flap was raised on a single, central, medial row perforator vessel, 3.2 mm in diameter, with no fat necrosis or delayed healing (Fig. 3).

A left symmetrising mastopexy and further fat transfer were performed in September 2012.

### Case 2

**Figure 4:**
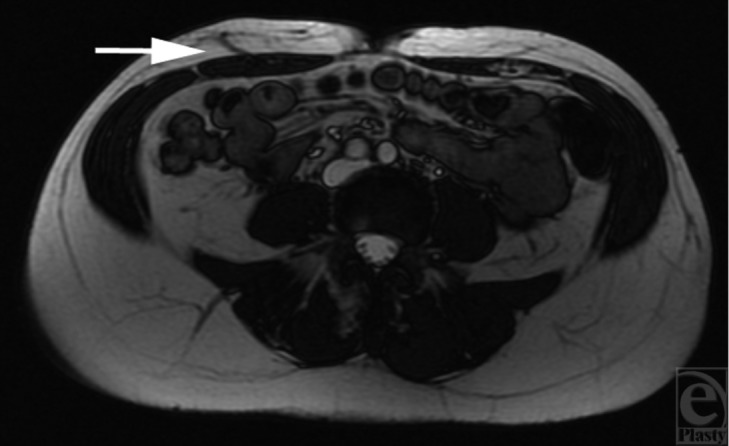
Case 2 - MR angiogram demonstrating a single large patent perforator below and right of the umbilicus (arrow).

**Figure 5:**
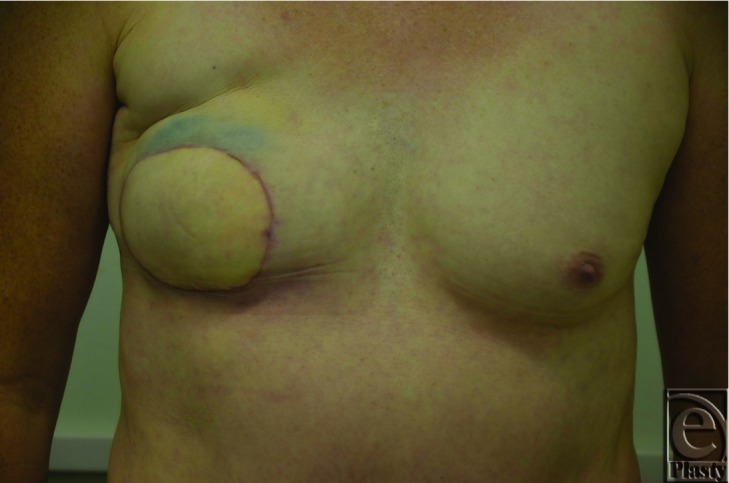
Case 2 - Appearance 1 week following DIEP flap breast reconstruction.

A 54-year-old woman underwent wide local excision and adjuvant radiotherapy for an invasive ductal carcinoma of the right breast in 2006. Sentinel lymph node biopsy was negative. The patient had no significant medical history and was an ex-smoker. Due to scar tethering and volume loss she underwent a series of five lipofilling procedures between December 2010 and December 2012. Each fat graft was harvested from the infraumbilical abdomen using Coleman's technique, with 100 to 160ml harvested at each procedure. The patient developed a recurrence of her breast cancer, confirmed by biopsy in May 2013. She underwent right mastectomy and immediate reconstruction with free DIEP flap in July 2013. Preoperative MRA of the abdomen (Fig. 4) demonstrated a single large patent perforator below and right of the umbilicus. Clinical findings correlated with the scan and the above perforator was raised with the flap. There were no abnormal intraoperative findings. The patient had an unremarkable recovery and was discharged 5 days postoperatively. No fat necrosis or wound dehiscence was detected at follow-up clinic appointments (Fig. 5).

## DISCUSSION

Outcomes of free flaps following abdominal liposuction are inconsistently reported in the literature. Issues impacting flap success are the patency of perforator vessels, the timing of reconstruction, the type of reconstruction (TRAM vs DIEP flap), number of perforators recruited into the flap and extent of previous abdominal surgery. Previous studies have focused on TRAM flaps in live patients^2^^,^^3^^,^^4^ or cadaveric^2^^,^^5^ or animal models.^6^ Studies assessing the effects of liposuction on DIEP flaps in clinical practice have followed a single episode of liposuction only.^6^^,^^7^

DIEP flaps are usually dependent on perfusion from one (sometimes two) perforator(s) while a TRAM flap utilises many or all regional perforators from the deep inferior epigastric artery. The success of TRAM flaps can therefore be expected to be more reliable than DIEP in patients with previous abdominal surgery.

Following liposuction, perforators may either be bruised and thrombosed, or hypothetically transected or avulsed. The size of liposuction cannula used, the volume of fat removed, and the use of tumescent technique may influence this damage.^3^ Thrombosed vessels have potential to recannalise, which may be more reliable than neovascularisation of transected vessels.

### Timing of surgery

Perforator vessels of the rectus abdominis muscle have potential for re-growth after undermining of the skin and fat of the anterior abdominal wall.

Ribuffo et al^2^ studied the perforator arteries of 10 patients following abdominoplasty using colour duplex scanning. They were found to regain their patency by 3 months, and increase in diameter up to 6 months post-op. However, new vessel calibre never exceeded 40% of preoperative diameter. In the same paper, a cadaveric study of a woman 10 years post-abdominoplasty showed no re-growth of accompanying veins. The authors caution against the use of TRAM flaps in post-abdominoplasty patients.

Hallock and Rice investigated the use of a pedicled TRAM flap after abdominoplasty in a rat model. 13.7% flap viability (Zone I only) was reported when raised 10 months after abdominoplasty.^8^

Emeri et al performed liposuction on two cadavers followed by microangiography of the deep inferior epigastric artery. The perforator vessels from the rectus fascia remained intact after liposuction.^5^ As cadaveric studies do not allow for the physiological response to vascular trauma that occurs in live patients,^1^ this should not necessarily be extrapolated to the clinical setting, but our findings suggest that this is probably the case.

Inceoglu et al^9^ studied the effect of liposuction on perforator vessels by Duplex ultrasound. 57.8% of abdominal perforators were not detected at 2 weeks or 3 months post-operatively. However, Salgarello et al showed ‘no significant injury to most perforating vessels’ at 6 months following superficial subdermal liposuction, as detected by colour and pulsed-wave Doppler sonography.^1^

Five DIEP flaps after single-episode liposuction were reported by DeFrene et al.^6^ The minimum interval between liposuction and reconstruction was 4 years. Our report demonstrates safe DIEP flap breast reconstruction 16 months following multiple liposuction procedures. Though there remains no agreement on a timeline for vessel recovery prior to safe free tissue transfer without complications, we believe six months should be the minimum interval.

### Imaging modality

Several imaging modalities have been used for the assessment of perforators prior to reconstruction.

Methylene blue dye^4^ gives a rapid assessment of the presence of perforators and the soft tissue territory supplied, but it does not identify which perforators are responsible for the perfusion observed. It may be difficult to judge the usefulness of the perforators present if appearance of the dye at the skin is delayed. There is also concern that dye may harm vessels by vasoconstriction secondary to inhibition of nitric oxide.

Duplex ultrasound^1^^,^^2^^,^^3^ allows the evaluation of perforator number, location, diameter and flow velocity. It does not give information on the precise tissue perfusion of a given perforator, which is relevant if there are concerns about smaller branches being injured during liposuction.

CT angiography accurately assesses perforator course, which aids the raising of flaps by reducing the potential for perforator injury.^7^ It also provides information on perforator size and number, allowing the surgeon to pre-select the optimum perforators for a given flap. We prefer magnetic resonance angiography as it provides the benefits of CTA described above without ionising radiation, and with a lower risk of reaction to intravenous contrast medium.

## SUMMARY

We use preoperative angiography of the abdominal wall for our DIEP patients and would particularly recommend it in cases of multiple previous abdominal liposuction. Patients should be counselled regarding a small potential increase in the risk of flap failure or partial flap necrosis.

We demonstrate that DIEP flaps can safely be raised following multiple liposuction procedures to the abdomen, and flaps can be raised successfully without perfusion-related complications.
